# Posterior uterine wall rupture in an unscarred uterus in a term pregnancy; a case report

**DOI:** 10.1016/j.crwh.2024.e00664

**Published:** 2024-11-09

**Authors:** Mesfin Ayalew Tsegaye, Alemayehu Nigusssie Adugna, Rebecca Haile Tesfay, Elias Gashaw Endegnanew, Kidist Nega Aragaw

**Affiliations:** a Dilla University, College of Medicine and Health Sciences, Department of Obstetrics and Gynecology, Dilla, Ethiopia; b Dilla University, College of Medicine and Health Sciences, Department of Internal Medicine, Dilla, Ethiopia

**Keywords:** Uterine rupture, Unscarred, Hysterectomy, Previous cesarean section, Bradycardia

## Abstract

Uterine rupture is a catastrophic separation of the uterine walls due to several risk factors. It is a common complication of scarred uterus during labor and delivery. Early detection is associated with better maternal and fetal outcomes. Due to nonspecific presentation, a high level of suspicion especially on pregnancies with risk factors could help pick uterine rupture early. This report presents a gravida 11 para 10 mother who presented with vaginal bleeding and severe abdominal pain after laboring for 24 h at home. Intra-op findings were approximately 1000 ml of hemoperitoneum with the fetus and the placenta floating on the peritoneal cavity and a 13 cm posterior uterine rupture with vaginal extension. A subtotal hysterectomy and left salpingo-oophorectomy were done She was discharged well after several blood transfusions. The objective is to present a rare case of posterior wall uterine rupture and to emphasize the importance of early detection of posterior wall uterine ruptures.

## Highlights

• Uterine rupture is a separation of all uterine walls due to several risk factors.
• Posterior wall uterine rupture is a very rare incident.
• Posterior wall rupture may present with nonspecific symptoms.
• Early detection and intervention reduce complications.
• A grand multi-para mother with posterior wall rupture was treated successfully.

## 1 Introduction

Uterine rupture, or the nonsurgical disruption of all uterine layers, is associated with maternal and perinatal morbidity and mortality [1]. Risk factors include previous cesarean section, multiparity, malposition, obstructed labor, breech extraction, uterine instrumentation and congenital uterine abnormalities [1,2]. It is more prevalent in developing countries, where it is associated with multiparity and obstructed labor. In contrast, in developed countries, it is usually a consequence trial of labor after previous cesarean delivery. [1,3]

Uterine rupture has no specific clinical presentation, which leads to delayed diagnosis and treatment. Fetal heart rate abnormalities such as bradycardia are the most common early presentation. Additionally, it may present with vaginal bleeding, cessation of uterine contractions, abdominal tenderness, and pain [1,[4], [5], [6]].

This case report describes the presentation and management of posterior uterine wall rupture in labor at term in a woman in her eleventh pregnancy.

## 2 Case Presentation

A 38-year-old woman, gravida 11 para 10, who had been amenorrheic for 9 months and had irregular antenatal care was referred in labor from a primary hospital because of suspected uterine rupture. She did not know the date of her last menstrual period and had not had an early ultrasound scan. Her previous pregnancies had resulted in uncomplicated spontaneous vaginal deliveries.

She had been laboring at home for 24 h before coming to hospital and presented with severe abdominal pain and bleeding.

Upon presentation, her blood pressure was within the normal range, but she was tachycardic (130/min) and her conjunctiva were pale. She had abdominal guarding and rigidity, and there were no uterine contractions. The fetus was palpable abdominally separate from the uterus. The cervix was 6 cm dilated with no fetal presenting part palpable. There was also tenderness on the fornices. No fetal heartbeat was detected.

**Fig. 1:**
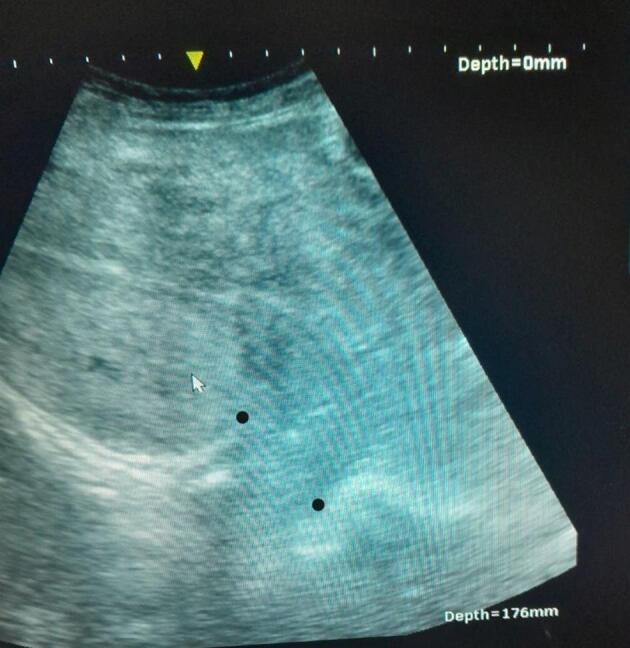
Posterior wall uterine rupture showing discontinuity on the posterior wall (the space between the two dark dots).

Investigations revealed a hemoglobin level of 6 g/dL. Otherwise, her liver and renal function tests were normal. An ultrasound scan showed fluid collection in the peritoneal cavity and an empty uterus with discontinuity in the posterior wall implying uterine rupture (Fig. 1).

**Fig. 2:**
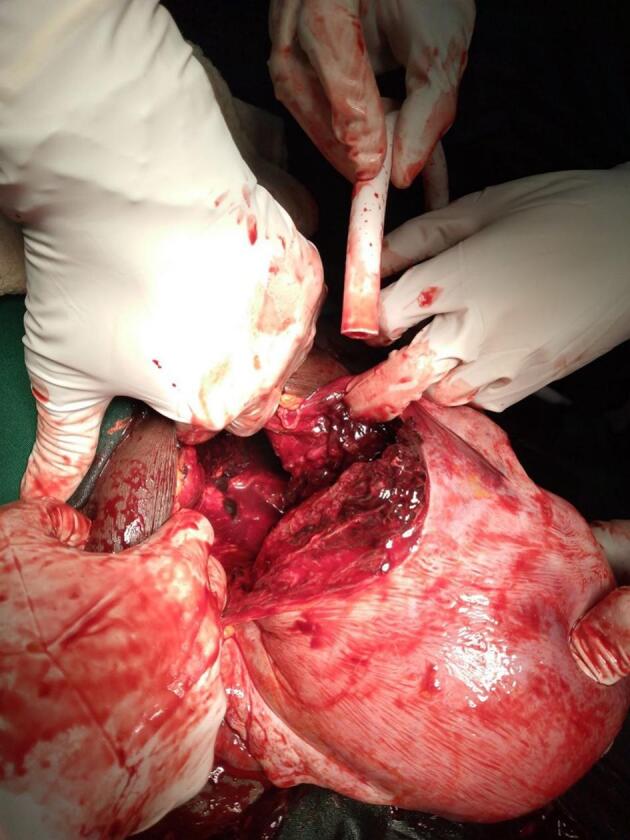
Posterior uterine wall rupture.

Surgery, using a vertical infra-umbilical incision, was undertaken after administration of intravenous fluids. Intraoperatively, approximately 1000 ml of blood was removed from the peritoneal cavity. A stillborn 2.9 kg fetus and placenta were extracted from the peritoneal cavity. There was a 13 cm posterior wall transverse uterine rupture at the lower uterine segment, with extension to the posterior vaginal wall (Fig. 2). Additionally, there was a left-sided broad ligament hematoma. Since the rupture was irreparable, a subtotal hysterectomy and left salpingo-oophorectomy were performed. Postoperatively, the patient received multiple units of blood transfusions and was discharged after 8 days.

## 3 Discussion

Uterine rupture is rare in an unscarred uterus: its incidence is about 0.5–2 per 10,000 deliveries, with most of them occurring in multiparous women. [2] It is around 50 times more prevalent in women with a scarred uterus [7]. It is called primary rupture if it occurs in an unscarred uterus and secondary rupture if it is on a uterus with a previous scar, malformation, or damage. [2]

Posterior wall ruptures are rare, constituting only 7.3 % of all uterine ruptures in one study [8]. Previous cesarean scar, previous uterine rupture, induction of labor, grand multiparity, unsupervised labor, and a short interpregnancy interval are some of the risk factors for uterine rupture. Some risk factors specifically associated with posterior wall rupture include previous procedures like curettage and myomectomy, congenital malformations of the uterus, fetal malposition and malpresentation, and abnormal placentation. These are thought to weaken the wall of the uterus, eventually leading to rupture. Presentation can be asymptomatic or accompanied by only mild symptoms. The risk factors for the patient described were multiparity and an unsupervised prolonged labor [2,4,8,9].

The rupture can extend transversely, obliquely, or vertically/longitudinally, based on the areas: transversely and obliquely in the cervical area and vertically/longitudinally in the broad ligament. It may also extend to the bladder. Depending on the size of the rupture, the fetus and the placenta may migrate out of the uterus if the presenting part of the fetus is not deeply engaged [10]. Once this happens, early intervention is important to reduce maternal and fetal morbidity and mortality. Fetal complications include neonatal admission to an intensive care unit, neonatal asphyxia, and intrauterine fetal death. The mother is also at risk of significant bleeding, which can lead to hemorrhagic shock, bladder injury, hysterectomy, and death [1,7,10]. In this case, the mother labored for around 24 h at home due to cultural practices, which delayed her arrival at a health facility. Thus, there is a need to improve antenatal care in low-resource settings and increase awareness of obstetric emergencies.

## 4 Conclusion

Education is required among healthcare professionals about the risk factors for uterine rupture. Early detection is critical as nonspecific signs and symptoms can delay diagnosis.
